# Future time perspective and academic procrastination among nursing students: The mediating role of mindfulness

**DOI:** 10.1002/nop2.1630

**Published:** 2023-02-14

**Authors:** Shuang Li, Jingwen Su, Di Zhao, Juan Wang, Gongchao Wang

**Affiliations:** ^1^ School of Nursing and Rehabilitation Shandong University Jinan China

**Keywords:** academic procrastination, future time perspective, mindfulness, nursing students

## Abstract

**Aim:**

To explore the relationship between university nursing students' academic procrastination, mindfulness, and future time perspective.

**Design:**

A cross‐sectional study.

**Methods:**

A total of 343 university nursing students recruited from eight provinces in China have reported procrastination characteristics through fulfilling an online website link. The main instruments involved Mindfulness Attention Awareness Scale (MAAS), Zimbardo Time Perspective Scale, and Procrastination Assessment Scale (PASS).

**Results:**

Participants who self‐assessed higher frequency and degree of academic procrastination tended to possess lower future time consciousness, and lower mindfulness. Mindfulness served as a mediation effect between future time perspective and academic procrastination. The study indicates that weakening an individual's procrastination can be alleviated through future time awareness and mindfulness. Concentrating on influencing factors, strengthening nursing student's future time perspective, and practicing mindfulness training could assist educators to decrease students' procrastination intentions and behaviours.

## 1 INTRODUCTION

Procrastination is a problem that plagues people from all walks of life. In particular, students are more likely to experience procrastination and fail to resolve by one's insufficient resources (Milgram & Toubiana, 1999). Procrastination refers to putting off the start or completion of a planned assignment even though expecting the negative consequences (Steel, 2007). Academic procrastination is quite widespread, with several researchers reporting that approximately 50%–95% of students habitually exposed to procrastination (O'Brien, 2002; Schouwenburg, 1992; Solomon & Rothblum, 1984). Moreover, the prevalence of the phenomenon appears to be on the rise (Steel, 2007). Procrastinate tasks that are required to perform, such as weekly reading assignments, reading for exams, and completing term papers (Ozer et al., 2009; Pychyl et al., 2000; Tice & Baumeister, 1997). In view of these challenges, a variety of students may suffer from incompletion of the assignments on time, poor academic performance (Ellis & Knaus, 1977; Grau & Minguillon, 2013; Klassen et al., 2008; Zarick & Stonebraker, 2009) and avoidance, anxiety, stress, and mental or physical health problems (Sirois, 2014; Spada et al., 2006; Stöber & Joormann, 2001; Tice & Baumeister, 1997). Recent evidence revealed that procrastination had a negative correlation with academic performance and the consequence of procrastination may inevitably be a low achievement; If a student put off writing homework and then submitting it, or delayed in preparing for an exam and failed to cover all relevant materials, the ensuing result seems inevitable to be poor academic performance (Kim & Seo, 2015). Future time perspective (FTP) is considered as a way that a person connects with the past, present, and future psychological thoughts, and a future‐oriented time frame constructed by individuals to make a general plan for the future (Zimbardo & Boyd, 1999). Results from several studies suggested that a variety of cognition concerning the past, present, and future time formed from different personal experiences, leading to a change of future time awareness. In this process, unconscious FTP will affect their information processing, decision‐making, and goal setting, and ultimately affect the individual's behaviour (Griva et al., 2015). With the characteristics of motivation, anticipation, and tendency, FTP is related to the individual's attitude, behaviour intention, and perceived behavioural control (Andre et al., 2018). Existing research recognized that varying in forms of procrastination will have diverse effects on the individual's time orientation. The higher the degree of future orientation, the lower threshold of perceived procrastination, but the positive correlation of future orientation to avoiding procrastination is not evident (Ferrari & Díaz‐Morales, 2007). Other research implied that whether past‐negative or past‐positive orientations are connected with behavioural procrastination (Gupta et al., 2012). To be precise, individuals with past negative tendencies are likely to postpone tasks, but the degree of procrastination is independent of past negative tendencies (Díaz‐Morales et al., 2008). Alternatively, a survey conducted by Sirois (2014) has proposed that the present‐oriented time perspective is positively correlated with procrastination. Although time perspective has closely linked with procrastination, the evidence that does exist about what higher procrastinators with present and past time perspective are obvious contradictions and controversies in the existing researches.

Considering the relevance between FTP and academic procrastination, it is necessary to explore the factors that have an effect on procrastination. Some previous findings indicated that mindfulness served as a key part when coping with emotional problems (Britton et al., 2012; Santarnecchi et al., 2014). Mindfulness involves not only the current environment in connection with their own experience but also capable to adjust and guide one's thoughts of the present (Kabat‐Zinn, 1994). Enhancing the attention and consciousness of current experience or reality is characterized as mindfulness (Brown & Ryan, 2003). With the ability to reduce psychological stress and change personal behaviour, mindfulness has been considered as a cognitive‐behavioural strategy (Ludwig & Kabat‐Zinn, 2008; Singh et al., 2008). Experiences of stronger initiative, more intensive pleasure sentiment, and less regularly disagreeable affection were closely relevant to mindfulness (Brown & Ryan, 2003). What is more, an increase in psychological well‐being and reduction of stress for individuals, attributed to a continuing mindfulness training course to deal with a variety of common stress and negative emotions (Goodman & Schorling, 2012; McGarrigle & Walsh, 2011). Motivating patients to link differently to their physical symptoms when fewer discomfort consequences or even associated with other medical disorders, mindfulness therapy alleviated the severity of anxiety and depression symptoms to a certain extent (Hofmann et al., 2010). Similarly, a recent study showed that negative emotions, such as anxiety and depression were effectively relieved through mindfulness‐based exercises (Desrosiers et al., 2013). It can be seen that reducing procrastination is inseparable from the level of mindfulness. Previous research indicated that FTP and academic procrastination were related. However, there have been no studies to explore whether mindfulness may impact a nursing student's academic procrastination with FTP.

Our study attempted to explore academic procrastination among university nursing students and its relationship with FTP and mindfulness and examine whether academic procrastination was associated with future time perspectives directly and indirectly, through the mediation of mindfulness. Consequently, this study proposed the hypothesis that mindfulness would mediate the negative relationship between FTP and academic procrastination.

## 2 METHODS

### 2.1 Participants

The convenience samples were university nursing students from eight provinces of China, who were recruited through a popular social application in China, WeChat. The cross‐sectional study was conducted in the form of an online website link, with data being gathered via students that may be interested. Criteria for selecting the respondents were as follows: (1) being a university nursing student; (2) volunteer to participate in the study; (3) completed the entire questionnaire as required. Students with mental illness or cognitive impairment and the response that were not logical were excluded. A questionnaire performed to obtain sociodemographic information and self‐reported measures of mindfulness, future time perspective, and academic procrastination. The Ethics Committee of the institution approved the study. All participants were informed about the purpose of the study and assured that confidentiality and anonymity would be maintained.

To make the multiple linear regression model stable, the sample size should be at least 10 times as large as the independent variables (Kendall, 1975). This study considered nine independent variables and the predicted response rate was approximately 70%. The required sample size was estimated to be greater than 129. Of the 380 individuals invited to participate, 343 completed the study questionnaire with a 90.3% response rate.

### 2.2 Measures

#### 2.2.1 Mindfulness

The Mindful Attention Awareness Scale (MAAS) was applied to measure the personal physical and psychological experience of being current state, which was composed of 15 items. Ranging from “always” (scoring one) to “never” (scoring six), a 6‐point Likert scale reported participants the extent to which they agreed with the statements. Scores are summed, and higher scores indicate greater levels of current awareness. Chen et al. (2012) completed the Chinese version of MAAS through translation, reliability, and validity test, and the scale used in the study possessed good construct validity (Cronbach's α = 0.89; test–retest reliability = 0.87). In this study, the Cronbach coefficient of the scale was 0.873.

#### 2.2.2 Future time perspective

We used the Chinese version of the General Future Time Perspective Scale compiled by Song (2004), based on the Zimbardo Time Perspective Scale (Zimbardo & Boyd, 1999). The 6‐point Likert scale comprised 20 items, with five dimensions (imagery, efficacy, or purpose consciousness concerning the future, behavioural commitment, and far‐target orientation), ranging from “strongly agree” (scoring one) to “do not agree” (scoring six). This scale was high reliability (Cronbach's α = 0. 852).

#### 2.2.3 Academic procrastination

To measure the procrastination of university students when performing various academic tasks, the first part of the Procrastination Assessment Scale (PASS) was used (Solomon & Rothblum, 1984). The 18‐item scale estimated a student's current delay in the academic environment, using a five‐point Likert scale from never (scoring one) to very often (scoring five). Six academic tasks were measured: attending meetings and completing academic tasks, accomplishing weekly reading assignments, finishing writing assignments, preparing for exams, and performing administrative tasks. The total score was positively associated with the degree of procrastination, and the more distress caused by academic procrastination. In this survey, the Cronbach's α for the PASS was 0.903.

### 2.3 Data analysis

Descriptive analysis of samples was reported as mean (standard deviation) and frequency (percentage). A Pearson correlation examined the relationships between mindfulness, FTP, and academic procrastination. PROCESS macro was performed to construct a mediation model using 5000 bootstrapping samples, while participants' characteristics, such as gender, educational background, grade, residence, an only child, major satisfaction, were included in the analysis as controlling variables (Hayes & Preacher, 2014). The total effects, direct effects, and indirect effects of the mediation model indicated statistically significant levels when the 95% confidence intervals (CIs) did not contain zero. Statistically significant level was set at a *p*‐value of 0.05. All analyses were carried out using SPSS (v. 25.0, SPSS Inc).

## 3 RESULTS

### 3.1 Participant characteristics

A total of 343 respondents, 249 university nursing students were female (72.6%). Among participants, more than two‐thirds of the participants were undergraduate students (78.4%), junior college and graduate students accounted for 11.4% and 10.2%, respectively. Of all samples, the second graders had the largest number of students (31.5%) followed by the first‐grade students (30.3%).Only 60 students (17.5%) of the total were satisfied with their major. For respondents, 233 (67.91%) were from cities and 132 (61.5%) were only children. Participant characteristics are given in Table 1.

**TABLE 1:** Participant characteristics.

Variable	*N*	%
Gender		
Male	94	27.4
Female	249	72.6
Educational background		
Junior college	39	11.4
Undergraduate	269	78.4
Graduate	35	10.2
Grade		
1	104	30.3
2	108	31.5
3	85	24.8
4	36	10.5
5	10	2.9
Residence		
Urban	233	67.9
Rural	110	32.1
Only child		
Yes	132	38.5
No	211	61.5
Major satisfaction		
Very satisfied	60	17.5
General	224	65.3
Not so satisfied	52	15.2
Very dissatisfied	7	2

### 3.2 Correlation analysis

The correlation coefficients for FTP, mindfulness, and academic procrastination are presented in Table 2. A positive correlation was found between mindfulness and FTP (*r* = 0.469, *p* < 0.01). There were negative correlations between mindfulness and the frequency of procrastination, the degree of procrastination, and academic procrastination (*r* = −0.399 to −0.504, *p* < 0.01). Furthermore, FTP was negatively associated with the frequency of procrastination, the degree of procrastination, and academic procrastination (*r* = −0.235 to −0.340, *p* < 0.05).

**TABLE 2:** Correlations among variables

Variables	Mean	SD	1	2	3	4	5
1Mindfulness	2.14	1.15	1				
2Future time perspective	1.54	0.73	0.469**	1			
3frequency of procrastination	2.28	0.63	−0.413**	−0.319**	1		
4degree to procrastination	2.06	0.75	−0.399**	−0.235*	0.280**	1	
5Academic procrastination	2.16	0.56	−0.504**	−0.340**	0.734**	0.854**	1

*Note*: *p < 0.05; **p < 0.01.

### 3.3 Mediation model

**FIGURE 1:**
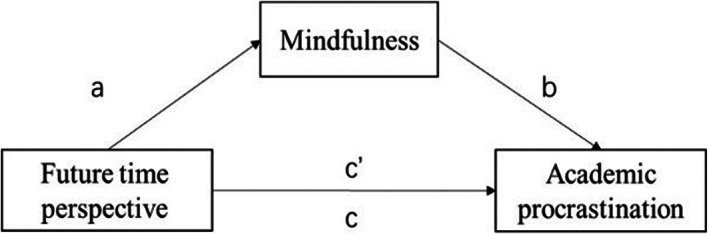
The mediation effect of mindfulness between future time perspective and academic procrastination.

The relationships of main variables are presented in Table 3 and Figure 1. Multiple linear regression analysis revealed that FTP was negatively associated with academic procrastination (β = −0.247, *p* < 0.001), and positively associated with mindfulness (β = 0.681, *p* < 0.001). In addition, both mindfulness and FTP were negatively related to academic procrastination with all demographic characteristics included as control variables (β = −0.212, *p* < 0.001; β = −0.102, *p* < 0.05). The results of bootstrapping method indicated that a direct effect exists between FTP and academic procrastination (β = −0.102, SE = 0.038, 95% CI = [−0.172, −0.026]), and a total effect of FTP on academic procrastination was −0.247 (SE = 0.041, 95% CI = [−0.327, −0.166]). Furthermore, the relationship between FTP and academic procrastination was partially mediated through mindfulness (ab = −0.144, SE = 0.024, 95% CI = [−0.196, −0.101]). The indirect effect accounted to the total mediation was 58.5%.

**TABLE 3:** Multiple linear regressions for the mediating effects of mindfulness on future time perspective and academic procrastination.

Regression equation		Goodness of fit		Significance		
Dependent variable	Independent variable	*R* ^2^	*F*	β	SE	*t*
Academic procrastination		0.135	6.453			
	Future time perspective			−0.247	0.0408	−6.045***
Mindfulness		0.243	13.425			
	Future time perspective			0.681	0.078	8.681***
Academic procrastination		0.278	14.232			
	Future time perspective			−0.102	0.041	−2.475*
	Mindfulness			−0.212	0.026	−8.147***

*Note*: *p < 0.05; **p < 0.01; ***p < 0.001.

## 4 DISCUSSION

Several studies had suggested that the prevalence of procrastination among students was high, it seems essential to explore the actual state in which students experience academic procrastination and closely identify the protective factors of procrastinators in the academic environment (Fernie et al., 2018; Guo et al., 2019; Klingsieck et al., 2013; Steel et al., 2018). Supposing that mindfulness might serve as a mediating factor, we verified the relationships between FTP, mindfulness, and academic procrastination among university nursing students. The data analysis illustrated that the academic procrastination levels of students were slightly lower than previously reported levels (Guo et al., 2019; Ozer et al., 2013; Zhang et al., 2018). The discrepancy might be attributed to over half of the participants were first and second graders in our study.

The current study found FTP potentially predicting academic procrastination. In other words, higher levels of FTP, lower the frequency and degree of procrastination, which agrees with previous findings (Denız et al., 2009; Ellis & Knaus, 1977). A recent study involving 335 university students showed that mindfulness could negatively predict academic procrastination (Jobaneh et al., 2016). Consistent with this view, a study interviewed 377 college students and identified that time perspective had a significant effect on procrastination (Kim et al., 2017). This finding further supports that university nursing students' problematic behaviours were inclined to occur without developed future time orientation, despite expected long‐lasting consequences. In a previous study, academic procrastination was found to be related to FTP by interviewing 1098 undergraduate students (Jin et al., 2019). That is, FTP was negatively linked to academic procrastination. With stronger FTP, university nursing students consequently tend to make judgements on future prospects in advance, better guide current behaviours, positive personal future behaviour, decrease delay intentions.

Individuals with low mindfulness are more likely to put off tasks, which causes a higher opportunity of anxiety or even severe negative emotions. We also found that the mindfulness of university nursing students negatively predicted behavioural procrastination, which is in line with previous researches (Goodman & Schorling, 2012; McGarrigle & Walsh, 2011). A study including 113 clinicians supported that mindfulness had a direct impact on a clinician's vitality (Martin‐Cuellar et al., 2021). Furthermore, existing research illustrated that less procrastination was inseparable from higher mindfulness because the mindfulness is positive, the individual's self‐evaluation (Kong et al., 2014). when encountering difficulties, individuals objectively evaluated one's abilities and difficulties, thereby avoiding fear and low self‐esteem. Of the factors that affect academic procrastination, inattention is a dispensable factor; Mindfulness motived a person to focus on the present, perceive all the feelings with a truthful and open mind (Xie et al., 2021).

On the basis of analysis, mindfulness was found to remain a partial mediating effect between FTP and procrastination. Researchers attempted to evaluate whether FTP had an impact on behavioral procrastination and described that individual with higher FTP was inclined to better control procrastination in mental and behavioral (Kim et al., 2017). As mindfulness provided positive psychological resources for university nursing students with FTP, the interaction effect of FTP and mindfulness may increase the individual ability to buffer mental problems. On the contrary, university nursing students with low mindfulness and less FTP had a poor ability to regulate impulsive emotions and behaviors. Results of the previous study indicated that for procrastinators, low mindfulness may contribute to pessimistic mood and endanger physical health (Sirois & Pychyl, 2013; Sirois & Tosti, 2012). Additionally, FTP was a personal spiritual resource, usually aroused by a person when encountering adversity and the individual also becalmed resilient (Seginer, 2008).

## 5 IMPLICATIONS AND LIMITATIONS

Academic procrastination affects the nursing students ‘academic performance and physical and mental health. Despite extensive researches on student procrastination, our study further confirms the mediation value of mindfulness and attempts to explore probable connections between FTP, mindfulness, and academic procrastination. The study indicated the stronger future time perspective may reduce academic procrastination by mindfulness. In view of these findings, it is beneficial for educational administrators to take targeted measures to enhance students’ FTP, and then help university nursing students to be conscious to cultivate personal cognition and thoughts concerning the future time, to establish a correct concept of time, cultivate interest in learning, improve learning efficiency, reduce academic procrastination behaviour. Furthermore, there is a global nursing staff shortage and a deepening economic recession. Therefore, it is necessary to train more nursing students. However, academic procrastination can cause a natural loss of students, prevent students from acquiring the knowledge and skills to provide quality care for patients, and are not conducive to an increase in the number and quality of nursing staff. Through mindfulness‐based training during daily routines helps create a more favourable learning environment, beneficial to training more high‐quality nursing students, but also can provide some reference for the mental health education of college students. University nursing students will critically perceive the future, be optimistic about the future, and take the initiative to reduce procrastination when understanding the role of future time perspective and mindfulness.

Nevertheless, our study also had limitations. This cross‐sectional study has failed to determine a causal relationship between FTP, academic procrastination, and mindfulness. Then, we merely recruited full‐time university students without part‐time university students, which lacked a full of understanding the state of procrastination among part‐time university students. In addition, we only reported Chinese college students, limiting the generalizability of the results. Notwithstanding these limitations, this study offers some insight into the mediation effect of mindfulness on future time perspective and academic procrastination, which might benefit educators to develop interventions with respect to decrease university nursing students' procrastination behaviour through future time perspective and mindfulness training. Further research could be undertaken to enrol a wide range of university nursing students around the world for perfecting the evidence.

## 7 ETHICS STATEMENT

The project was approved by our Institutional Review Board (Shandong University). All participants were informed about the purpose of the study and assured that confidentiality and anonymity would be maintained.

## Data Availability

The datasets are available from the corresponding author on reasonable request.
